# Association Between Childhood Exposure to Pet Cats and Later Diagnosis of Schizophrenia: A Case-Control Study in Saudi Arabia

**DOI:** 10.7759/cureus.32401

**Published:** 2022-12-11

**Authors:** Ramzi M Hakami, Ahmed A Alnaami, Khaled A Shbeeli, Atheer Y Al Suhaym, Bashaer H Khormi, Ibrahim H Faqihi, Ibtihal H Hadi, Khalid M Kulaybi, Salihah I Mawkili

**Affiliations:** 1 Psychiatry, Eradah Mental Health Complex, Jazan, SAU; 2 Geriatric Psychiatry, Eradah Mental Health Complex, Jazan, SAU; 3 Emergency Department, Eradah Mental Health Complex, Jazan, SAU

**Keywords:** toxoplasma gondii, saudi arabia, toxoplasmosis, schizophrenia, cat ownership

## Abstract

There is an increasing number of reports suggesting an effect of adverse environmental factors during vulnerable periods of prenatal and perinatal development in the etiology of schizophrenia. Cat-transmitted infections, especially *Toxoplasma gondii*, are possible risk factors for the later development of schizophrenia. We conducted a case-control study to examine childhood cat ownership in 78 patients with schizophrenia (cases), 78 outpatients with depression and anxiety disorders (control group one), and 78 outpatients with no psychiatric history (control group two). Cat ownership before the age of 13 was reported by 52.6%, 44.9%, and 24.4% of patients in cases, control group one, and control group two, respectively. Compared with non-psychiatric patients, patients with schizophrenia were 3.4 times more likely to report owning cats in their childhood (odds ratio (OR)=3.441; p=0.000; 95% confidence interval (CI)=1.740-6.804). Compared with both control groups, the likelihood of owning a cat as a child was 2.1 times more among cases (OR=2.093; p=0.008; 95% CI=1.203-3.640). Age, female gender, and family history appeared to be positively associated with cat ownership and schizophrenia. This study supports the evidence of a relationship between childhood exposure to pet cats and the later development of schizophrenia. Further in-depth research is needed to clarify the relationship between exposure to pet cats in childhood and later diagnosis of schizophrenia, adjusting for potential confounders.

## Introduction

Schizophrenia is a chronic, pervasive neuropsychiatric disorder characterized by marked hallucinations, delusions, and disturbance in thought, emotion, perception, and behavior. Schizophrenia is diagnosed by exclusion of other causes of psychosis after obtaining a thorough psychiatric history and examination. The pathophysiology of schizophrenia is not fully understood owing to its relative complexity and heterogeneity. Given that schizophrenia occurs increasingly within families, genetic factors have been suggested to play a major role in its etiology, and some candidate predisposing genes were identified [1,2]. In addition, environmental factors have been shown to contribute to the development of schizophrenia. Traditionally, nearly 1% of people are affected by schizophrenia [3]. However, the global estimates of schizophrenia are variable, depending on population characteristics and research methodologies.

In the past two decades, studies on infectious pathogens as a possible cause of schizophrenia have increased [4]. Such cases have been hypothesized to be caused by different kinds of infections, including polioviruses [5], rubella virus [6], herpes simplex virus type 2 [7], and influenza virus [8]. Furthermore, some studies suggested a relationship between the later development of schizophrenia and postnatal exposure to viruses and bacteria that cause meningitis and encephalitis [9]. Most of the attention, however, has been directed at *Toxoplasma gondii* (*T. gondii*), an obligate intracellular neurotropic parasite that infects approximately one-third of the global population [10].

Given the vast array of infections that may increase susceptibility to schizophrenia, we focused this study on childhood exposure to specific pathogens that can be transmitted primarily by house cats. Felines (i.e., cats) are the only complete definitive host for *T. gondii*, which can be transmitted from cats to humans through direct or indirect contact (e.g., through drinking water contaminated with oocysts or eating undercooked meat containing tissue cysts) [4].

Human toxoplasmosis is usually asymptomatic in immunocompetent individuals but can produce a wide range of severe clinical symptoms in immunocompromised ones. Psychiatric manifestations such as hallucination and delusion are frequent in some cases of adults with acute toxoplasmosis [11]. 

The mechanism by which an infection causes psychiatric symptoms remains uncertain. A unifying hypothesis by Kinney et al. proposes that during vulnerable periods of prenatal and perinatal development, adverse genetic and environmental factors cause abnormalities in the development and function of the immune and nervous systems, which in turn become risk factors for developing schizophrenia later in life [12]. Consistent with this hypothesis, several complementary studies on monkeys, rats, and humans have found that prenatal exposure to psychosocial maternal stress and malnutrition has significant effects on the immune system function and thymus structure in adolescents and adults [13,14]. Therefore, it is possible that prenatal and perinatal exposure to infections may impact the immune system resulting in neurotransmitter imbalance in subjects with schizophrenia [15]. Interestingly, in two randomized double-blind studies, Müller et al. found that patients with acute schizophrenia showed significantly greater improvement in response to the anti-inflammatory drug celecoxib given as an adjuvant to the antipsychotic drug risperidone, compared with patients given risperidone only. The authors concluded that immune dysfunction is related to the pathological mechanism of schizophrenia [16,17]. 

Five previous studies have examined the relationship between childhood cat ownership and the later development of schizophrenia, and four of them found a positive association [18-22]. The present study investigated childhood cat ownership as a possible risk factor for schizophrenia in the Saudi population.

## Materials and methods

Study population

This was a case-control study of patients with schizophrenia admitted to the inpatient units of Al-Amal Complex for Mental Health (currently named Eradah Complex for Mental Health). The hospital is the largest and only governmental hospital specializing in psychiatry in Jazan province, southwestern Saudi Arabia. A probability sample of 80 cases (38 males and 40 females) diagnosed with schizophrenia was selected. Two control groups were included. The first group was composed of 78 patients (38 males and 40 females) visiting the outpatient unit of the same hospital. Their diagnosis included depression (41 patients) and anxiety disorders (37 patients). The second group consisted of 78 patients with no psychiatric history (39 males and 39 females) admitted to different inpatient units of the King Fahad Central Hospital (KFCH). This is the largest central hospital in the Jazan province.

The inclusion criteria were as follows: (a) a formal diagnosis of schizophrenia (for cases), (b) a formal diagnosis of depression and anxiety (for psychiatric controls), and (c) no formal diagnosis of any psychiatric disorder (for non-psychiatric controls). The exclusion criteria were as follows: (a) a formal diagnosis of schizophrenia (for both control groups) or other psychiatric disorders (for non-psychiatric controls), (b) refusal to participate in the study, and (c) critical illness. Only two patients with schizophrenia were excluded because they were critically ill and refused to participate. None of the controls were excluded on the basis of the aforementioned exclusion criteria. The schizophrenia group and two control groups were matched for gender and age. Participation was completely anonymous. All participants were informed about the aim of the study, and verbal consent was obtained from each of them. The study was approved by the Institutional Review Board of Jazan University.

Data collection and processing

All participants were interviewed by the study authors between March and August 2017. Psychiatric patients were interviewed by five trained coauthors, one of them a consultant psychiatrist, and the others were psychiatry residents and medical students trained for the purpose of this study. Non-psychiatric patients were interviewed by two medical students in their final year of medical school with special training by a consultant prior to data collection. As a strategy to reduce ascertainment bias, interviewers were instructed to ask about the exposure of interest (cat ownership) at the end of the interview. 

A structured questionnaire was used, which included information about the subjects' age, residence, education, marital status, medical history, family history of psychiatric disorders, and family cat ownership before the age of 13 years. To assess cat ownership, participants were asked the following question: "To the best of your knowledge, did you or your family own a cat before you were 13 years of age?". In addition, patients with schizophrenia were asked about their diagnosis and treatment. Psychiatric diagnosis was made based on the International Classification of Diseases 10th Revision (ICD-10) [23]. 

## Results

Table 1 shows a comparison of sociodemographic characteristics of schizophrenia patients with those of controls (i.e., patients with depression and anxiety disorders, and patients with no psychiatric history). The mean age of patients with schizophrenia, other psychiatric diagnoses, and non-psychiatric patients was 42.38 years (standard deviation (SD) 14.01, range 19-72), 39.55 years (SD 13.61, range 19-86), and 40.62 years (SD 13.91, range 17-70), respectively. In all groups, the majority were rural, married, and unemployed (including students and retired individuals). Most of the study subjects were educated for 12 years or less (i.e., illiterate, primary, secondary, or high school), with higher rates of illiteracy reported by subjects with schizophrenia (28.2%). There were no significant differences between the 78 subjects with schizophrenia (cases) and 156 matched controls in any of the sociodemographic characteristics examined (Table 1).

**Table 1:** Sociodemographic characteristics of study groups (N=234)

Characteristics	Schizophrenia (n=78)	Depression and anxiety (n=78)	Non-psychiatric (n=78)
Gender			
Male	38 (48.7%)	38 (48.7%)	39 (50.0%)
Female	40 (51.3%)	40 (51.3%)	39 (50.0%)
Age (years)			
≤24	6 (7.7%)	8 (10.3%)	11 (14.1%)
25-34	18 (23.1%)	20 (25.6%)	16 (20.5%)
35-44	23 (29.5%)	25 (32.1%)	23 (32.1%)
45-54	12 (15.4%)	15 (19.2%)	10 (12.8%)
55-64	10 (12.8%)	6 (7.7%)	11 (14.1%)
≥65	9 (11.5%)	4 (5.1%)	5 (6.4%)
Residence			
Rural	51 (65.4%)	50 (64.1%)	40 (51.3%)
Urban	27 (34.6%)	28 (35.9%)	38 (48.7%)
Marital status			
Single	28 (35.9%)	25 (32.1%)	18 (23.1%)
Married	40 (51.3%)	41 (52.6%)	56 (71.8%)
Divorced/widowed	10 (12.8%)	12 (15.4%)	4 (5.1%)
Education			
≤ 12 years	60 (76.9%)	43 (55.1%)	60 (76.9%)
> 12 years	18 (23.1%)	35 (45.9%)	18 (23.1%)
Job status			
Employed	27 (34.6%)	32 (34.4%)	28 (35.9%)
Unemployed	51 (65.4%)	46 (59.0%)	50 (64.1%)

The percentage of cat ownership in patients with schizophrenia, controls with depression and anxiety disorders, and controls with no psychiatric history was found to be 52.6%, 44.9%, and 24.4%, respectively. The difference between patients with schizophrenia and non-psychiatric patients was highly significant, with schizophrenic patients being 3.4 times more likely to report owning cats in their childhood (odds ratio (OR)=3.441; p=0.000; 95% confidence interval (CI)=1.740-6.804). However, no significant difference was found between patients with schizophrenia and the control group with depression and anxiety disorders (OR=1.361; p=0.337; 95% CI=1.203-3.640). Patients with schizophrenia were significantly (2.1 times) more likely to report owning cats in their childhood than were both control groups (OR=2.093; p=0.008; 95% CI=1.203-3.640) (Table 2).

**Table 2:** Distribution of cat ownership before age 13 years in patients with schizophrenia (n=78) and controls with depression and anxiety disorders, and controls with no psychiatric history (n=156) ^1^ The three study groups were dichotomized as 0 (schizophrenia) and 1 (pooled controls; psychiatric and non-psychiatric patients)

Paired groups		Cat ownership before age 13	OR	p-value	95% CI
	Schizophrenia	41 (52.6%)	1.361	0.337	(1.203-3.640)
	Depression and anxiety	35 (44.9%)			
	Schizophrenia and	41 (52.6%)	3.441	0	(1.740-6.804)
	Non-psychiatric patients	19 (24.4%)			
	Schizophrenia and	41 (52.6%)	2.093	0.008	(1.203-3.640)
	Pooled controls^1^	54 (34.6%)			

The percentage of childhood cat ownership was highest in females with schizophrenia representing 60.0%, and lowest in females with no psychiatric history representing only 10.3% (χ2=21.355; p=0.000) of the respective groups. More males with depression and anxiety (47.4%) reported owning a cat in their childhood than did males with schizophrenia (44.7%) (χ2=0.053; p=0.818) (see Tables 3 and 4).

**Table 3:** The distribution of cat ownership before age 13 according to the gender and age in schizophrenia patients (n=78) and controls with depression and anxiety disorders (n=78)

Characteristics	Schizophrenia	Depression and anxiety	χ^2^	p-value
Gender				
Male	17/38 (44.7%)	18/38 (47.4%)	0.053	0.818
Female	24/40 (60.0%)	17/40 (42.5%)	2.452	0.179
Age (years)				
≤24	1/6 (16.7%)	1/8 (12.5%)	0.049	0.825
25-34	8/18 (44.4%)	7/20 (35.0%)	0.354	0.552
35-44	9/23 (39.1%)	9/25 (36.0)	0.05	0.823
45-54	7/12 (58.3%)	9/15 (60.0%)	0.008	0.93
55-64	8/10 (80.0%)	5/6 (83.3%)	0.027	0.869
≥65	8/9 (88.9%)	4/4 (100.0)	0.481	0.488

**Table 4:** The distribution of cat ownership before age 13 according to gender and age in schizophrenia patients (n=78) and controls with no psychiatric history (n=78)

Characteristics	Schizophrenia	Non-psychiatric	χ^2^	p-value
Gender				
Male	17/38 (44.7%)	15/39 (38.5%)	0.312	0.576
Female	24/40 (60.0%)	4/39 (10.3%)	21.355	0
Age (years)				
≤24	1/6 (16.7%)	3/11 (27.3%)	0.243	0.622
25-34	8/18 (44.4%)	1/16 (6.2%)	6.349	0.012
35-44	9/23 (39.1%)	7/25 (28.0)	0.668	0.414
45-54	7/12 (58.3%)	4/10 (40.0%)	0.733	0.392
55-64	8/10 (80.0%)	3/11 (27.3%)	5.383	0.016
≥65	8/9 (88.9%)	1/5 (20.0%)	6.644	0.01

Among schizophrenic patients, the frequency of childhood cat ownership was proportional to age, reaching 88.9% in the oldest subjects (≥65 years) and only 16.7% in the youngest (≤24 years). Similarly, all oldest subjects and only 12.5% of the youngest subjects with depression and anxiety reported owning cats in their childhood. Among patients with no psychiatric history, the rate of reported childhood cat ownership was highest in middle-aged adult patients reaching 28.0% and 40.0% in patients aged 35-44 and 45-54 years, respectively. Significant differences were found between patients with schizophrenia and non-psychiatric patients in the age groups 25-34 years (χ2=6.349; p=0.012), 55-64 years (χ2=5.383; p=0.016), and ≥65 years (χ2=6.644; p=0.010), with schizophrenia patients reporting higher rates of childhood cat ownership (Table 4).

We studied relevant family history of psychiatric disorders as a factor that might influence the association between cat ownership in childhood and the later development of schizophrenia, depression, or anxiety. The results showed that the percentage of cat ownership in childhood was higher among psychiatric patients with positive family history than was among those with negative family history. Cat ownership was reported by 64.7%, 62.5%, and 77.8% of patients with schizophrenia, depression, and anxiety, respectively, who had at least one first- or second-degree relative with the same disease. The difference was significant between patients with anxiety (χ2=6. 841; p=0.009). These results suggest that family history has an important role in predisposition to schizophrenia, depression, and anxiety, which may include childhood cat ownership as well as genetic and environmental factors influencing the association of childhood cat ownership with the above psychiatric disorders (Table 5).

**Table 5:** The distribution of cat ownership before age 13 in relation to relevant family history in schizophrenia patients (n=78) and controls with depression and anxiety (n=78)

Paired groups	Cat ownership before age 13	χ^2^	p-value
Schizophrenia with +ve family history	22/34 (64.7%)	3.564	0.059
Schizophrenia with −ve family history	19/43 (43.2%)		
Depression with +ve family history	10/16 (62.5%)	1.977	0.160
Depression with −ve family history	10/25 (40.0%)		
Anxiety with +ve family history	7/9 (77.8%)	6.841	0.009
Anxiety with −ve family history	8/28 (28.6%)		

## Discussion

Schizophrenia is a major public health problem with a destructive impact on patients, families, and societies. So far, there is no certain pathophysiology of schizophrenia, and the current paradigm in psychiatry is that schizophrenia is multifactorial, with both genetic, environmental, and psychosocial factors likely playing an important role in its etiology [24]. Recently, an increasing body of research has focused on the association between exposure to household pet cats in infancy and childhood and a later diagnosis of schizophrenia, with some authors finding positive associations [18-22] and others not [25]. A large population-based study conducted in Finland revealed that having a pet cat as a child was associated with an increased rate of adult schizotypal traits [25], especially among adults who had experienced cat bites as children [26]. Consistently, we found a high percentage of childhood cat ownership in patients with schizophrenia (52.5%) compared with patients with depression and anxiety disorders (44.8%) and patients with no psychiatric history (24.3%) (OR=2.093; p=0.008). The difference was highly significant between patients with schizophrenia and non-psychiatric controls (OR=3.441, p=0.000), but not significant between patients with schizophrenia and psychiatric controls (OR=1.361, p=0.337).

Pet cats are not common pets in Saudi Arabia, but stray cats are commonly seen and may represent a risk factor for toxoplasmosis. In a recent survey conducted in Riyadh, Saudi Arabia, the authors examined the sociodemographic characteristics of pet owners and showed that 49.5% reported a family member at risk of pet-related disease. The most common pet owned by Saudis in the study was cats, representing as much as 77.4% [27]. Cat ownership has been linked to human toxoplasmosis in several studies [10], although a few other studies found no association [28]. Possible explanations for assuming a causal relationship between toxoplasmosis and schizophrenia are summarized by Torrey et al. [4]. First, many studies have reported a higher prevalence of antibodies to *T. gondii *in individuals with schizophrenia than in controls [29-32]. Second, some adults with toxoplasmosis develop psychotic manifestations similar to those seen in schizophrenics [33]. Third, both conditions have epidemiological similarities in familial aspects and sociodemographic status. Fourth, antipsychotics have been shown to inhibit *T. gondii* replication and reduce dopamine levels [34,35]. Fifth, elevated dopamine, commonly seen in schizophrenics, has been found in experimentally infected animals [36].

Although not significant, the rate of cat ownership in our study was more in females with schizophrenia than in males. Previous studies on the gender difference in pet ownership have found that females were significantly more likely than males to own a cat, a dog, or both [37]. However, in both control groups with no psychiatric history, male patients reported higher rates of cat ownership than female patients. Moreover, gender was a significant factor in our results when we compared females with schizophrenia with females with no psychiatric history. This may indicate that the risk of developing schizophrenia is higher in females who owned a cat in their childhood than in males who owned a cat in their childhood, but more studies on this gender difference are necessary for a definitive conclusion.

Consistent with the heterogeneous nature of psychiatric disorder etiology [12], we found that family history has an important role in the predisposition to schizophrenia, depression, and anxiety. The majority of psychiatric patients who reported childhood cat ownership in this study also reported having at least one relative with the same condition. Therefore, future studies are recommended to examine the relationship between schizophrenia and early exposure to cats adjusting for well-known risk factors of schizophrenia, such as family history. More research is needed to find out whether this association is evident across populations. We recommend future research to provide more precise information on the timing of exposure to better characterize the risk of cat exposure in infancy and childhood in patients with schizophrenia.

This study had several limitations. Data regarding cat ownership were self-reported and thus may have been subject to inaccuracy. A collateral history was taken to confirm patients' responses when applicable. Given the observational nature of the study, we could not assess a possible causal relationship between exposure to cats in childhood and schizophrenia. Furthermore, the number of cats owned by subjects' families, duration of cat ownership, and history of cat-related injuries in childhood were not assessed. Finally, the study included only patient subjects; therefore, the findings may not apply to the general population. 

## Conclusions

The findings of the present study add to an increasing number of reports suggesting an association between childhood cat ownership and the later development of schizophrenia. Patients with schizophrenia were 3.4 times and 2.1 times more likely to report owning a pet cat as children compared to non-psychiatric controls and pooled controls including psychiatric patients with depression and anxiety. These associations could be caused by a combination of sociodemographic, neurological, immunological, and other biological factors. The study has many limitations, and the reported associations may be coincidental and need to be examined in the study context. In light of the findings of this study, more in-depth research is required to determine the probable causes behind these associations. Understanding the mechanisms underlying the early exposure to pet cats and later diagnosis of schizophrenia may shed light on how environmental exposures function as important factors in the etiology of schizophrenia and other psychotic disorders and inform the design of therapeutic approaches.
